# The sentinel tree nursery as an early warning system for pathway risk assessment: Fungal pathogens associated with Chinese woody plants commonly shipped to Europe

**DOI:** 10.1371/journal.pone.0188800

**Published:** 2017-11-29

**Authors:** Anna Maria Vettraino, Hong-Mei Li, Rene Eschen, Carmen Morales-Rodriguez, Andrea Vannini

**Affiliations:** 1 DIBAF-University of Tuscia, Viterbo, Italy; 2 CABI, Chinese Academy of Agricultural Sciences, Beijing, China; 3 CABI, Delémont, Switzerland; Brigham Young University, UNITED STATES

## Abstract

Introduction of and invasion by alien plant pathogens represents the main cause of emerging infectious diseases affecting domesticated and wild plant species worldwide. The trade in living plants is the most common pathway of introduction. Many of the alien tree pathogens recently introduced into Europe were not previously included on any quarantine lists. To help determine the potential risk of pest introduction through trading of ornamental plants, a sentinel nursery was established in Beijing, China in 2008. The sentinel nursery planting included four of the most common ornamental woody species shipped to Europe including *Ilex cornuta* var. *fortunae*, *Zelkova schneideriana*, *Fraxinus chinensis* and *Buxus microphylla*. Symptoms developing on these species within the sentinel nursery were detected in 2013 and consisted of necrotic spots on leaves, canker and stem necrosis, shoot blight and shoot necrosis. Fungi associated with the trees and their symptoms included *Alternaria alternata* detected from all hosts; *Diaporthe liquidambaris* and *Diaporthe capsici* from bark and leaf necrosis of *Zelkova schneideriana*; *Botryosphaeria dothidea* and *Nothophoma quercina* from stem cankers on *Fraxinus chinensis* and leaf necrosis on *Ilex cornuta*; and *Pseudonectria foliicola* from leaf necrosis on *Buxus microphylla*. Next generation sequencing analysis from asymptomatic tissues detected eighteen OTU’s at species level among which some taxa had not been previously recorded in Europe. These results clearly demonstrate that looking at trees of internationally traded species in the region of origin can reveal the presence of potentially harmful organisms of major forestry, landscape or crop trees. Results of this study also provide an indication as to how some disease agents can be introduced using pathways other than the co-generic hosts. Hence, sentinel nurseries represent one potential mechanism to address the current lack of knowledge about pests in the countries from where live plants are shipped and the threats they represent to native flora and crops in importing countries.

## Introduction

Introduction and invasion by alien plant pathogens represent the main cause of emerging infectious diseases (EIDs) affecting domesticated and wild plant species worldwide [1]. An analysis of temporal invasion patterns has demonstrated how the number of EIDs increased exponentially in the last two centuries, and that this trend is primarily associated with living plants and plant commodity trading [1]. The history of EIDs of woody plants, in natural and domesticated environments, confirms the trade of living plants as the most common pathway of introduction. Of the more than 125 taxa listed in the European Database of the Invasive Forest Pathogens (IFPs) (https://www.eustafor.eu/eu-projects/isefor/), including fungi and oomycetes, 54 (43%) are believed to have been introduced through trading of exotic living hosts. Furthermore, most of these EIDs were introduced unnoticed and described once they became invasive [2]. Classical examples are chestnut blight caused by *Cryphonectria parasitica* in the USA; Dutch elm disease caused by *Ophiostoma ulmi* and *O*. *novo ulmi* in Europe; stain canker of sycamore caused by *Ceratocystis platani* and cypress canker caused by *Seiridium cardinale*. Most recent examples are sudden oak death caused by *Phytophthora ramorum* both in Europe and in USA [3]; alder decline caused by *Phytophthora alni*; ash dieback caused by *Hymenoscyphus fraxineus* [4] and root and butt rot of conifers caused by *Heterobasidion irregulare* in Europe [5]. Most of these pathogens become invasive as the result of novel host—pathogen interactions, which often involve a new host species (host jump) or different populations of the same host. The ‘vector’ host plant is frequently taxonomically related to the new host in the invaded environment. For example, the chestnut blight fungus *C*. *parasitica* was introduced in the USA with trade in Asiatic chestnut species. However, this cannot be considered the rule. The cause of the oak powdery mildew *Erysiphe quercicola*, of probable Asiatic origin, and already recorded in oak forests in France associated with flag-shoot symptoms, was possibly introduced through host shift from traded exotic host species such as *Quercus phillyraeoides* [6]. However, Kirshner and Liu [7] reported anamorphic *E*. *quercicola* on two tropical hosts commonly traded to Europe as ornamentals, *Cinnamomum camphora* and *Murraya paniculata* highlighting the risk of host jump from taxonomically distant species. Furthermore, because of co-evolution, pathogens introduced with native hosts are not expected to produce striking symptoms [3] that are detectable in quarantine routine inspections. In fact, woody plants harbour in the different tissues and organs, a variable number (up to several hundreds) of endophytic microorganisms that do not cause symptoms. Some of these are beneficial or indifferent symbionts, others are potential pathogens occurring in latency in the native host plants. The ash fungal pathogen *H*. *fraxineus* was known to be non-pathogenic in its native range, Far East Asia, but its introduction into Europe has led to widespread dieback of the native European ash species *Fraxinus excelsior* and *Fraxinus angustifolia*, with which it had not co-evolved [8]. The above examples highlight the difficulty in predicting and preventing the spread of EIDs caused by alien invasive plant pathogens (AIPP).

Recently the sentinel tree concept was developed in order to provide an early warning system to detect potential AIPPs before they move from their place of origin [9] [10]. Two main strategies apply to the sentinel tree concept: the sentinel plantations and the sentinel nurseries. Sentinel plantations include the use of species native to a specific geographic area (e.g. Europe), and planted in an exotic environment (e.g. Asia). Trees in these plantations are exposed to the pressure of resident pests and pathogens and should display symptoms. Roques et al. [9] and Vettraino et al. [10] have recently demonstrated the efficacy of the sentinel plantations in describing novel host-parasite interactions for insects and pathogens, respectively. Sentinel nurseries are collections of woody species from a specific geographic region (e.g. Asia) selected among the most traded to another geographic region (e.g. Europe). Sentinel nurseries are established in the exporting country, in nurseries or open fields, and routinely inspected for their resident microbial community. Assuming that native-to-native host-pathogen interactions might not result in obvious expression of symptoms [3], appropriate diagnostic methods must be employed in order to detect even the latent, unnoticed microbes and, specifically, those organisms with the potential of being pathogenic to the flora found in the importing country. In this context, the sentinel nurseries represent a new tool to detect potential pathogenic organisms for which Pest Risk Analysis (PRA) can be utilized. This could then lead to the identification of appropriate mitigation measures before the introduction of microbes.

This paper reports the first example of microbial community assessment of a sentinel nursery established in Beijing (China) with four of the most commonly traded woody ornamental species to Europe.

## Materials and methods

The sentinel nursery was established at the Shunyi district, Beijing (40°14′27”N 116°49′47”E) in 2008. Four woody plants species were selected among the live plants commonly imported from China to Europe at the beginning of the experiments: *Ilex cornuta* var. *fortunei* (Lindl.) S.Y.Hu, *Buxus microphylla* Siebold & Zucc, *Fraxinus chinensis* Roxb. and *Zelkova schneideriana* Hand.-Mazz. The nursery was established in April 2012 with plants bought in the same exporting nursery. Since their purchase, plants were not protected against potential pests. Five blocks of 20 plants were planted for each species, i.e. 100 plants per species. No plant was taller than 80 cm, to correspond to classical exported plants. The distance between plants was 50 cm and between blocks 1.5 to 2 m. The nursery was regularly inspected in 2012 and 2013 for presence of symptoms and signs of diseases. In May 2013, samples were collected from symptomatic tissues observed on the four species. Samples for next generation sequencing (NGS) analysis were collected from healthy tissues (leaves and bark) of 5 randomly chosen individuals (one per block) representing each species. Samples from each plant species were bulked and each bulk sample was placed in a single plastic bag and sent to the laboratory. DNA extraction from host tissues and fungi were carried out in China at the CABI-Ministry of Agriculture joint laboratory in Beijing.

### Fungi isolation and molecular phylogenetic identification

Samples for isolation were surface sterilized, (60 s in 75% ethanol, 3 min in 3% NaClO, and 30 s in 75% ethanol), rinsed in sterile distilled water and aseptically cut into square fragments not exceeding 5x5 mm. Fragments were plated onto Petri dishes containing potato dextrose agar (PDA, Oxoid, UK, 39 g l ^-1^) amended with streptomycin sulphate (0.06 g l^-1^), and incubated at 25 ± 2°C. A hyphal tip of each fungal morphotype was sub-cultured on fresh media from each distinct colony.

Genomic DNA was extracted from fungal colonies using the Qiagen DNeasy PlantMini Kit (Qiagen, The Netherlands) according to the manufacturer’s instructions. DNA was quantified using the Qubit dsDNA HS Assay Kit (Thermo Fisher, USA) and sent to Italy at DIBAF-University of Tuscia for molecular identification. The rDNA ITS1-5.8s-ITS2 (ITS) region was amplified using the primer pair ITS-1 and ITS-4 [11]; the glyceraldehyde-3-phosphate dehydrogenase (gpd) gene was amplified with primers gdp1 and gdp2 [12]. Amplicons were purified with NucleoSpin Gel and PCR Cleanup (Mackery Nagel, Germany).

Sanger sequencing was performed by Macrogen Europe Laboratory (The Netherlands). Forward and reverse sequences were assembled and edited using BioEdit (Ibis Bioscience, CA, USA) and compared with the NCBI database (https://blast.ncbi.nlm.nih.gov). Sequences of the isolates and those of related taxa, downloaded from the GenBank database, were aligned using pair-wise CLUSTALW alignment [13]. Phylogenetic analysis for ITS, and concatenated ITS and gdp alignments were conducted with the dataset reported in the S1 Table. These alignments were assembled automatically in CLUSTALW with manual editing. Neighbour joining analysis, based on K2P distances and tested by 1000 bootstrap replicates, were conducted for each alignment to confirm the identification of query sequence to the genus or species level, using PHYLIP 3.6 [14]. Trees were drawn using TreeView [15]. Bayesian inference analysis was conducted with MrBayes v. 3.1 [16], applying a general time reversible (GTR) substitution model with gamma (G) and proportion of invariable site (I) parameters to accommodate variable rates across sites. Two independent runs of Markov Chain Monte Carlo (MCMC) using 4 chains were run over 1,000,000 generations. Trees were saved each 1,000 generations, resulting in 10,001 trees. Burn-in was set at 50,001 generations (i.e., 51 trees), well after the likelihood, values converged to stationary, from the remaining trees the consensus trees and posterior probabilities were calculated.

### Characterization of the fungal community by NGS analysis

Samples for NGS analysis were not surface sterilized. Genomic DNA was extracted from samples as described above. The non-coding nuclear rDNA ITS region was amplified using the fungal primer pair ITS1F (5’-AxxxCTTGGTCATTTAGAGGAAGTAA- 3’) and ITS2 (5’-BGCTGCGTTCTTCATCGATGC- 3’), where A and B represent the two pyrosequencing adaptors (CCATCTCATCCCTGCGTGTCTCCGACTCAG and CCTATCCCCTGTGTGCCTTGGCAGTCTCAG) and xxx the sample identification barcoding key. The following PCR conditions were used: 94°C for 4 min, 30 cycles of 30 s at 94°C (denaturation), 50°C for 1 min (annealing) and 72°C for 90 s (extension), followed by 10 min at 72°C. Positive and negative controls were included in the PCR. Amplicons were purified using the Agencourt AMPure XP system (Beckman Coulter Inc., USA) and quantified with the Qubit dsDNA HS Assay Kit. The library was sequenced in 1/16 of a standard PicoTitre Plate with the 454 Life Sciences GS-FLX System (Macrogen).

### Bioinformatics and statistical analyses

Raw data were trimmed and analysed using the software CLOTU [17]. Sequence reads with undetermined nucleotide, with any mismatch against tags and primers, and shorter than 150 bp were removed from the data set. Sequence reads from each sample were clustered into similarity-based OTUs (operational taxonomic units) using BLASTClust (single linkage clustering) at 97% similarity and 75% overlap in sequence length between reads in the pairwise alignments [18] [19].

Representative sequences from each OTU were submitted to BLASTn [20] for comparison against the GenBank non-redundant (NCBI-nr) database.

OTUs were only assigned to species level if, i) the query sequence matched database sequences from fungal isolates (including at least one vouchered specimen) with E-values ≤e−100 and percentage sequence identity ≥97%, and ii) at the lowest E-values, when there were no contradictions among different species within the same genus [21]. At lower percentage sequence identity, the query read was identified to genus (identity 95–96%) family (identity 90–94%) order (identity 80–88%) and class level (identity <80%).

Blast results were inspected manually to remove inconsistencies. Sequences were not considered if there was a contradiction in genus assignment in the reference database as we suspected misidentification. Phylogenetic trees were built as described above.

To assess sampling efficiency, a rarefaction curve was generated by means of the Analytic Rarefaction 1.3 software [22], using 10 specimens as a step parameter for calculation. Statistical analysis of data and generation of heatmaps were carried out with Prism version 7.00 software (GraphPad Software, California, USA) and Systat 11 (Systat Software, Inc.).

## Results

### Fungal isolation and molecular identification

**Fig 1:**
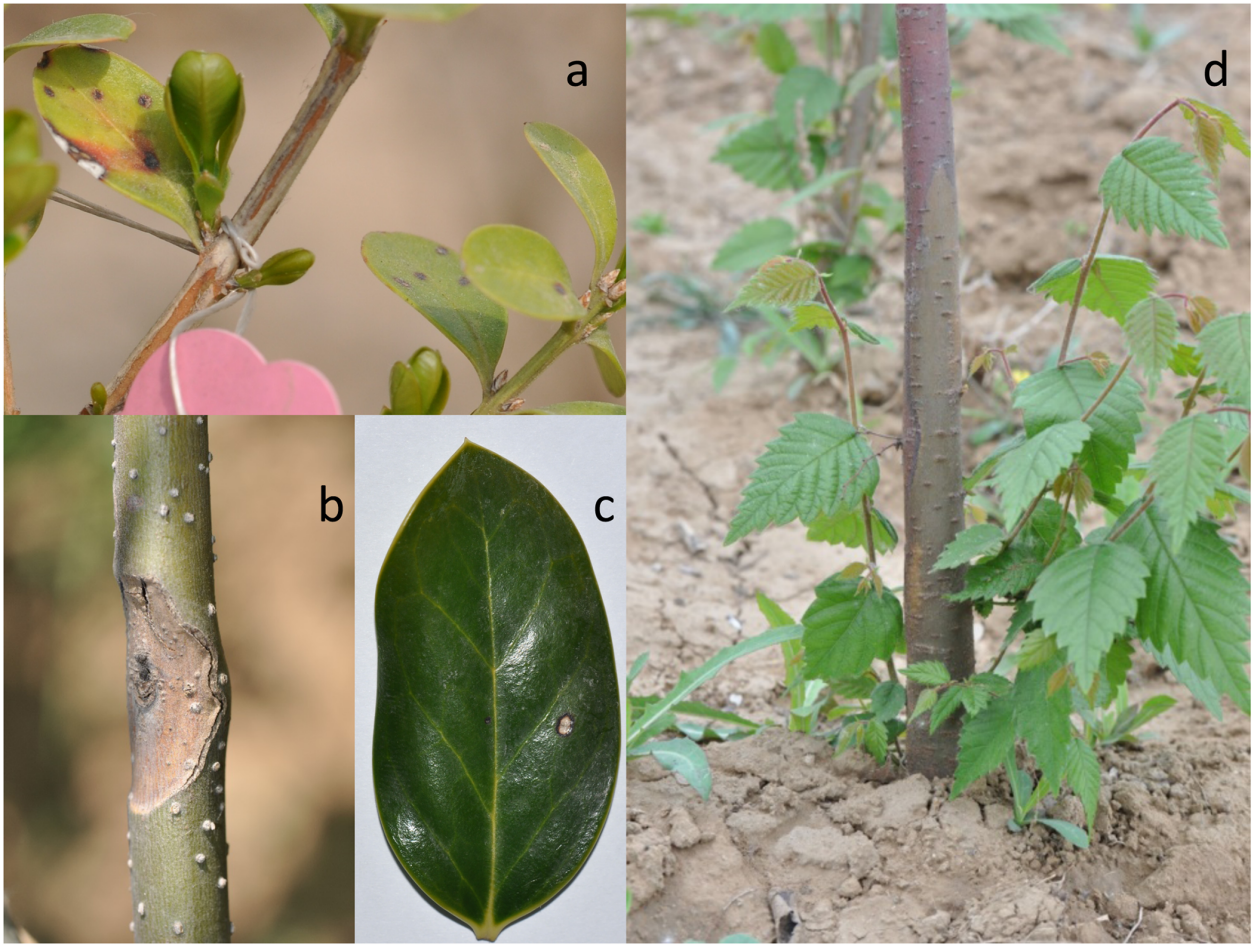
Symptoms on sentinel nursery trees. Leaf necrosis on *Buxus microphylla* (a); canker on *Fraxinus chinensis* (b); leaf necrosis on *Ilex cornuta* var. *fortunei* (c); and bark necrosis on *Zelkova schneideriana* (d).

Symptoms detected in 2013 consisted of necrotic spots on leaves of *B*. *microphylla* (Fig 1a) and *I*. *cornuta* (Fig 1c), and canker and stem necrosis on *F*. *chinensis* and *Z*. *schneideriana* (Fig 1b and 1d). Shoot blight and shoot necrosis were recorded on *F*. *chinensis* and *I*. *cornuta*, respectively.

**Fig 2:**
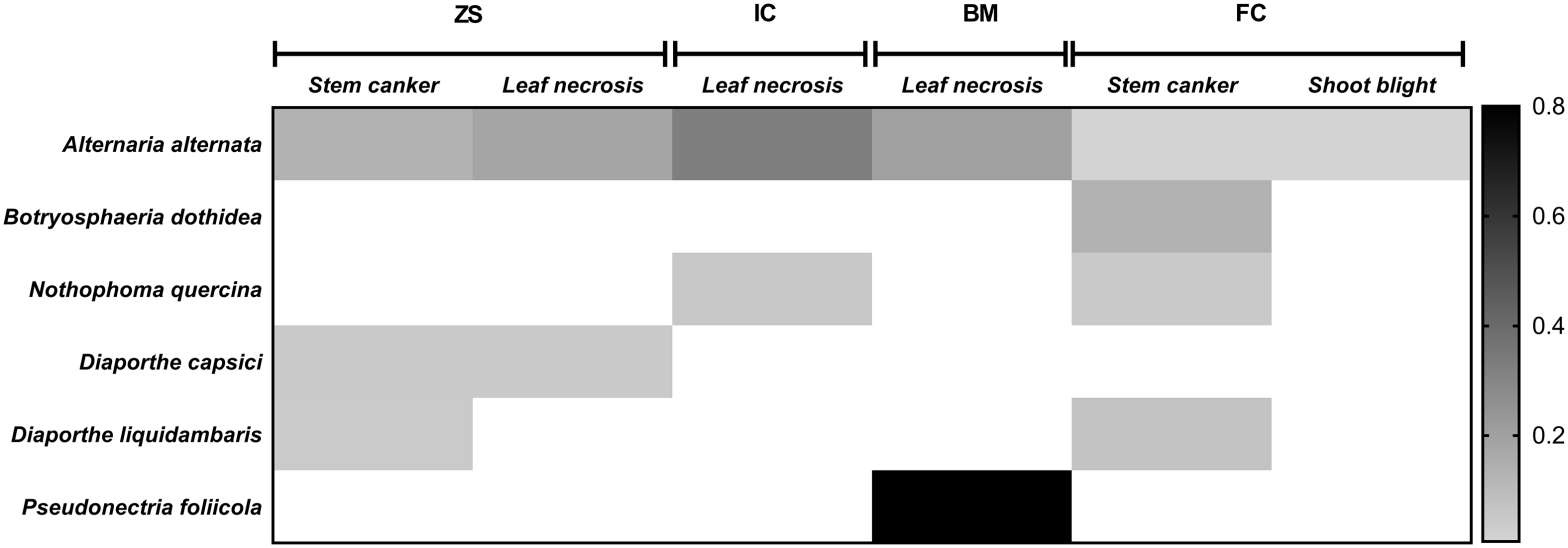
Heatmap of the abundance of fungal species identified by culturing analysis from symptomatic host tissues. Darker colour indicate a higher abundance of the isolates. *Zelkova schneideriana* (ZS); *Ilex cornuta* var. *fortunei* (IC); *Buxus microphylla* (BM); *Fraxinus chinensis* (FC).

Over 276 tissue fragments were plated and 146 (about 52%) produced fungal colonies in culture. Six different morphotypes were assigned to species level based on culture characteristics and molecular traits. *Alternaria alternata* (Fr.: Fr.) Keissl. was found on all hosts; *Diaporthe liquidambaris* (C.Q. Chang, Z.D. Jiang & P.K. Chi) Udayanga & Castl. and *Diaporthe capsici* Punith. were isolated from bark and leaf necrosis of *Z*. *schneideriana*; *Botryosphaeria dothidea* (Moug.: Fr.) Ces. & De Not. and *Nothophoma quercina* (Syd. & P. Syd.) Q. Chen & L. Cai were isolated from stem cankers on *F*. *chinensis* and from leaf necrosis on *I*. *cornuta*; and *Pseudonectria foliicola* (L. Lombard & Crous) was recovered leaf necrosis on *B*. *microphylla*. The relative abundance and distribution on hosts of the six species is shown as heatmap in Fig 2.

**Fig 3:**
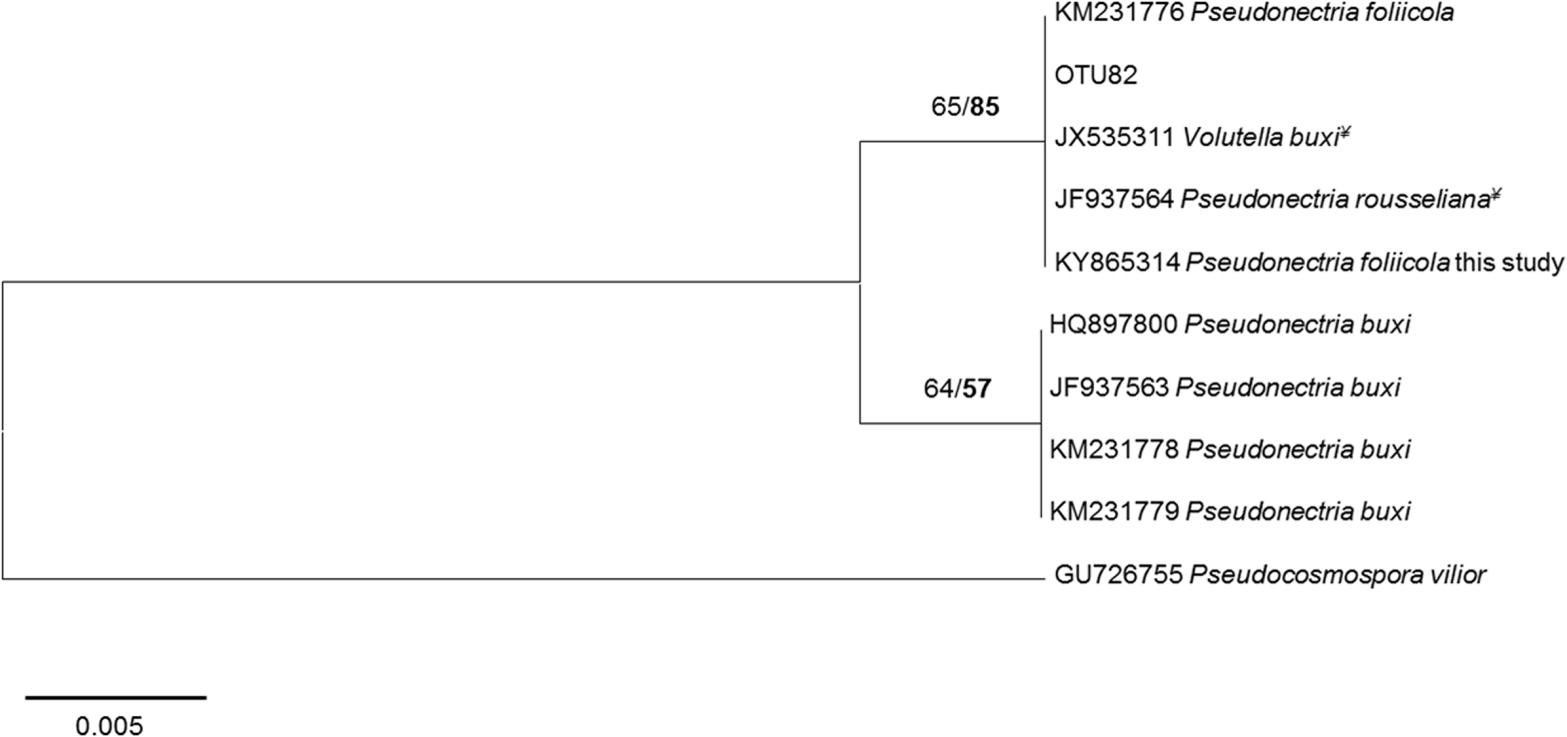
Neighbour-joining trees of the OTU82 (from NGS analysis), the ITS sequence of isolate of *Pseudonectria foliicola* from this study, and top BLAST hits species (with GenBank accession numbers). Numbers above branches represent bootstrap support for the nodes and posterior probability based on Bayesian analysis of the dataset (in bold). ^*¥*^ Sequence with 100% of identity with *P*. *foliicola*.

Representative sequences for each species were edited and deposited in GenBank with the accession numbers KY865308-KY865310, KY865312-KY865314, and KY947551 (S1 Table). Phylogenetic trees based on ITS (*D*. *liquidambaris*, *D*. *capsici*, *N*. *quercina* and *B*. *dothidea*) and concatenated ITS and gdp (*A*. *alternata*) are available as supporting information (S1 Fig). A phylogenetic tree including sequence of *P*. *foliicola* from this study and OTU82 from NGS analysis was developed (Fig 3). *Pseudonectria foliicola* was the only fungal species detected with both isolation and NGS analysis.

### Characterization of the fungal community by NGS analysis

**Fig 4:**
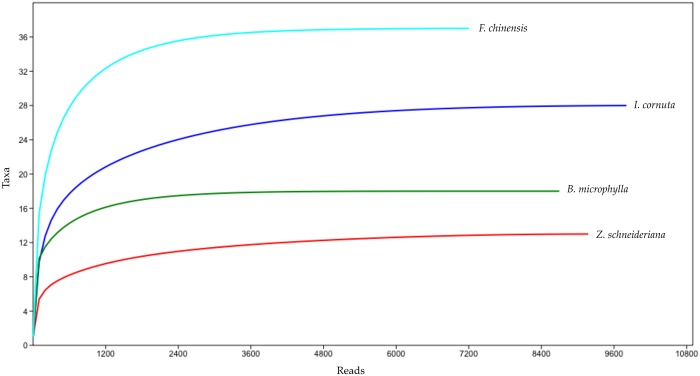
Rarefaction curve of the OTUs from each of the four sampled species.

After the quality filtering and removal of singletons, which are considered mostly artefacts [18], 35.872 reads were clustered in 106 OTU’s. The majority of OTU’s were Ascomycota (76 OTUs, 33.184 reads), followed by Basidiomycota (28 OTUs, 2.676 reads). Other taxa (Entomophthoromycota and Glomeromycota) were represented by less than 0.03% of the sequences (one OTU each). Rarefaction curves constructed for the data separated by host type showed saturated curves of the fungal community, suggesting that most of the biodiversity of the samples was inspected (Fig 4). Sequences of the 106 OTU’s were submitted to GeneBank under the accession numbers MF555485—MF555590.

Eighteen OTU’s (17,0%) were resolved at species level (Tab. 1), 80 at genus level, two at family level, one at order level, three at class level, one at subdivision and one at division level.

Pleosporales, specifically, the genus *Alternaria*, dominated the fungal community (58% of the total reads, 18 OTUs). The number of OTU’s for each host species was 56, 48, 25, 24 for *F*. *chinensis*, *I*. *cornuta*, *B*. *microphylla* and *Z*. *schneideriana*, respectively.

Phylogenetic trees of the OTU’s resolved at species level are shown in S2 Fig.

**Fig 5:**
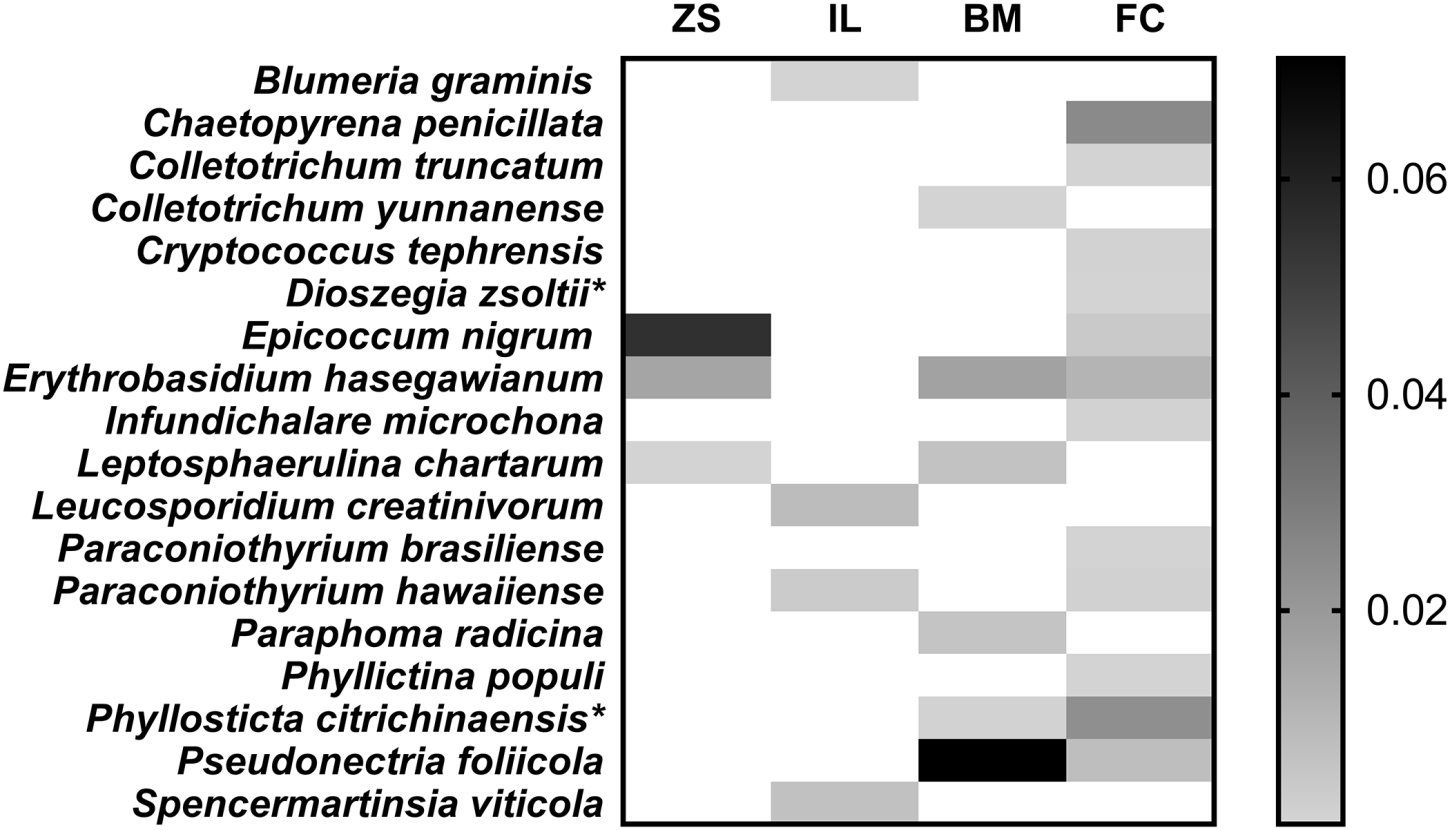
Heatmap of the abundance of the fungal taxa identified as species by NGS analysis from sentinel nursery plants. Higher intensities of the colour reveal higher abundances of the isolates. *Zelkova schneideriana* (ZS); *Ilex cornuta* var. *fortunei* (IC); *Buxus microphylla* (BM); *Fraxinus chinensis* (FC).* Ambiguous species identification, possible new species.

Taxonomic position, lifestyle, occurrence and recorded hosts/substrate for each of the eighteen fungal species are described in Table 1. The heatmap of the fungal species identified by NGS is showed in Fig 5.

**Table 1:** Taxonomic position, lifestyle, occurrence and recorded hosts/substrate for each of the eighteen fungal species identified by NGS analysis.

	Fungus		Host				Lifestyle	Occurrence	Reported host/substrate	Ref.
OTU	Species	Order	ZS	IC	BM	FC				
15	*Blumeria graminis*	Erysiphales	**-**	**+**	**-**	**-**	Plant pathogen	cosmopolitan	Poaceae	[23]
23	*Chaetopyrena penicillata*	Pleosporales	**-**	**-**	**-**	**+**	Plant pathogen	China, Middle East, South Africa	*Elaeagnus angustifolia*, *Medicaga sativa*, *Ephedra intermedia*	[23]
27	*Colletotrichum truncatum*	Glomerellales	**-**	**-**	**-**	**+**	Plant pathogen	cosmopolitan	broad-host range	[23]
28	*Colletotrichum yunnanense*	Glomerellales	**-**	**-**	**+**	**-**	Endophyte	China	*Buxus* spp.	[23]
37	*Cryptococcus tephrensis*	Tremellales	**-**	**-**	**-**	**+**	Yeast	unknown	different substrates	[26]
37	Dioszegia zsoltii*	Tremellales	**-**	**-**	**-**	**+**	Yeast	unknown	*Sapindus delavayi*, *Oryza sativa*, *Parthenocissus* sp., *Nerium indicum*	[27]
39	*Epicoccum nigrum Link*.	Pleosporales	**+**	**-**	**-**	**+**	Endophyte/weak pathogen	cosmopolitan	broad-host range	[23]
40	*Erythrobasidium hasegawianum*	Erythrobasidiales	**+**	**-**	**+**	**+**	Yeast	unknown	different substrates	[24]
47	*Infundichalara microchona*	Helotiales	**-**	**-**	**-**	**+**	Saprothroph	Netherland	*Pinus* litter	[23]
51	*Leptosphaerulina chartarum*	Pleosporales	**+**	**-**	**+**	**-**	Endophyte/weak leaf pathogen	cosmopolitan	broad-host range	[23]
53	*Leucosporidium creatinivorum*	Leucosporidiales	**-**	**+**	**-**	**-**	Yeast	unknown	different substrates	[25]
64	*Paraconiothyrium brasiliense*	Pleosporales	**-**	**-**	**-**	**+**	Endophyte	China, South Africa, Brazil	*Prunus* spp., *Coffee arabica*, *Acer truncatum*, *Phoradendron perrottetii*, *Tapirira guianensis*	[23]
65	*Paraconiothyrium hawaiiense*	Pleosporales	**-**	**+**	**-**	**+**	Endophyte	China, Hawaii	*Sophora chrysophylla*, *Acer truncatum*	[23]
66	*Paraphoma radicina*	Pleosporales	**-**	**-**	**+**	**-**	Saprophyte	cosmopolitan	*Allium cepa*, *Heterodera glycines*, *Malus sylvestris*, *Prunus cerasus*, *Solanum lycopersicum*	[23]
78	*Phyllictina populi*	Erysiphales	**-**	**-**	**-**	**+**	Plant pathogen	China, Korea	*Populus* spp.	[23]
80	Phyllosticta citrichinaensis*	Botryisphaeriales	**-**	**-**	**+**	**+**	Plant pathogen	China	*Citrus* spp.	[23]
82	*Pseudonectria foliicola*	Hypocreales	**-**	**-**	**+**	**+**	Plant Pathogen	New Zealand, North America	*Buxus* spp.	[23]
94	*Spencermartinsia viticola*	Botryisphaeriales	**-**	**+**	**-**	**-**	Plant pathogen	Asia, Africa, Europe, North America	broad-host range	[23]

* indicates OTU’s forming well-supported clade with isolates of the species but clustering separately.

Among these species, several were cosmopolitan or already recorded in Europe: the powdery mildew *Blumeria graminis* (DC.) Speer pathogenic to Poaceae, detected on *I*. *cornuta* (OTU15); *Colletotrichum truncatum* (Schwein.) Andrus & W.D. Moore from *F*. *chinensis* (OTU27), cause of anthracnose, blight and rot on multiple genera in multiple families, and particularly aggressive to *Jatropha curcas* (Euphorbiaceae) [23]; the broad host range endophytic fungus, *Epicoccum nigrum* Link., detected on *Z*. *schneideriana* and *F*. *chinensis* (OTU39), *Leptosphaerulina chartarum* Cec. Roux detected on *Z*. *schneideriana* and *B*. *microphylla* (OTU51); the saprophyte *Paraphoma radicina* (McAlpine) Morgan-Jones & J.F. White detected on *B*. *microphylla* (OTU66). OTU94, from *I*. *cornuta*, formed a monophyletic clade with the plant pathogen *Spencermartinsia viticola* (A.J.L. Phillips & J. Luque) A.J.L. Phillips, A. Alves & Crous recorded in China, South Africa, Europe, North America and Australia [23]. OTU23 from *F*. *chinensis*, was identified as *Chaetopyrena penicillata* (Fuckel) Höhn., previously recorded in China, Russia, Romania and South Africa as saprothroph on several substrates, and in Iran associated with dry rot of fruits of Russian olive (*Elaeagnus angustifolia* L.) [23].

Four Basidiomycota yeasts were also detected and their distribution is difficult to assess, *Erythrobasidium hasegawianum* Hamam., Sugiy. & Komag. [24] from *Z*. *schneideriana* (OTU40), *B*. *microphylla* and *F*. *chinensis*; *Leucosporidium creatinivorum* (Golubev) V. de Garcia, M. A. Coelho, T. Maia, L. H. Rosa, A. M. Vaz, C. A. Rosa, J. P. Sampaio, P. Goncalves, M. R. van Broock and D. Libkind [25] from *I*. *cornuta* (OTU53); *Cryptococcus tephrensis* Vishniac [26] (OTU36) and a possible new species of *Dioszegia* (OTU37) from *F*. *chinensis*. OTU37 formed a well-supported clade with *D*. *zsoltii* s.l. Bai, Takashima & Nakase [27], although, within the clade, clustered separately.

OTU78 and OTU38, from *F*. *chinensis* and *B*. *microphylla* respectively, were identified as the powdery mildew *Phyllactina populi* (Jacz.) Y.N. Yu., and the Glomerellales *Colletotrichum yunnanense* Xiao Ying Liu & W.P. Wu, both recorded in China only [23].

Two endophytic *Paraconiothyrium* species, *P*. *brasiliense* Verkley (OTU64), recorded in China, South Africa and Brazil, and *P*. *hawaiiense* (Crous) Damm, Crous & Verkley (OTU65), recorded in Hawaii and China [23], were both detected on *F*. *chinensis* and, the latter, *Paraconiothyrium hawaiiense* was also found on *I*. *cornuta* in this study.

*Infundichalara microchona* (W. Gams) Réblová & W. Gams was detected on *F*. *chinensis* (OTU47). This species, previously recorded in Netherlands, is a saprothroph on decaying bark and wood of *Pinus sylvestris* [28]

OTU79 from *B*. *microphylla* and *F*. *chinensis* formed a well-supported clade with isolates of *Phyllosticta citrichinaensis* X.H. Wang, Chen, Huang, Zhang, K.D. Hyde & H.Y. Li from *Citrus* although, within the clade, clustered separately (S2 Fig). Identity in NCBI with *P*. *citrichinaensis* sequences of isolates from *Citrus* was 97%.

OTU82 from *B*. *microphylla* and *F*. *chinensis*, formed a monophyletic clade with *P*. *foliicola* sequences on NCBI and with the ITS sequence of *P*. *foliicola* isolates from this study (Fig 3).

## Discussion

The results of the present paper represent the first example in application of the ‘sentinel nursery’ concept to Asiatic ornamental woody species commonly sold to Europe. Although several constraints in the approach have been highlighted and need to be resolved, the data presented clearly demonstrate the potential of the approach in identifying threatening fungal taxa in the region of origin before they are traded to Europe. Here we used two different detection strategies in order to describe the fungi associated with plant species commonly traded: i) classical biological detection through isolation from symptomatic tissues, and ii) molecular detection adopting throughput sequencing protocols in order to describe the fungal community associated with symptomless tissues.

### Classical detection techniques

The first strategy offers the advantage of having pure cultures of the putative pathogens with accurate specificity in identification, although sensitivity might not be satisfactory. In this study small leaf necroses were found on *B*. *microphilla* that were associated with *P*. *foliicola*, a specific leaf pathogen of *B*. *sempervirens* recorded in New Zealand and the United States [29]. However, its pathogenicity on other *Buxus* species is unknown at present. The species is new to China and not yet recorded in Europe. However, two ribosomal DNA sequences deposited in NCBI in 2012, JF937564.1 and JX535311.1, referring to *P*. *buxi* from China and Belgium, respectively (Fig 3), show 100% identity with *P*. *foliicola*, suggesting that the species is already present in Europe and the symptoms possibly confused with those of *P*. *buxi*. *Pseudonectria foliicola* was also recorded in NGS analysis in this study, with 634 reads from healthy *Buxus* tissues, suggesting a real risk of unnoticed introduction.

*Nothophoma quercina* was found associated for the first time with cankers on *F*. *chinensis* (Oleaceae) and leaf necrosis in *I*. *cornuta* (Aquifoliaceae). This species of fungus is known to cause cankers, shoot blight, leaf spot on several woody species in different parts of the world, including another Oleaceae species, *Olea europea*, in Spain and Tunisia; *Quercus* sp. (Fagaceae) in Ukraine [30]; *Chaenomeles sinensis* (Rosaceae) in Korea [31]; *Ziziphus jujuba* (Rhamnaceae) Mill. in China [32]; and *Pistacia vera* (Anacardiaceae) in Arizona [29]. Based on the increasing number of records reported in the literature in the last 5 years [30] [31] [32] [33], *N*. *quercina* must be considered a polyphagous cryptogenic pathogen whose geographic distribution is expanding and therefore has the potential of affecting a number of woody species.

Equally relevant is the isolation of *D*. *liquidambaris* from bark of *Z*. *schneideriana* (Ulmaceae). *Diaporthe liquidambaris* is an Asianic endophytic species found in branches of *Liquidambar formosana* (Altingiaceae) [34] and a pathogen of fruits of different woody hosts in China, including, *Tamarindus indica* (Fabaceae), *Psidium guajava* (Myrtaceae), and *Lansium domestricum* (Meliaceae) [35]. Its endophytic behaviour would facilitate its accidental introduction with traded asymptomatic plant stocks.

In this study, the widespread necrotic pathogen *B*. *dothidea* was found associated with cankers on *F*. *chinensis*. This is the first report of this polyphagous pathogen on this host.

### Next-generation sequencing

The NGS technology applied to the tree species in the sentinel nursery provided several advantages in discovering potential pathogens, but this technology is not without limitations. In this study, a ‘priming’ approach with fungal ITS primers was used to obtain amplicons for pyrosequencing. The use of an rDNA marker was obligatory, since rDNA gene clusters are the most represented sequences for fungi in online databases. However, these regions are not always capable of separating species. Furthermore, fungi are among the less represented phyla in the molecular database and it has been estimated that only a small percentage of the existing species is described [36]. Therefore, the identification of several OTU’s remain ambiguous or unresolved in this study. These OTU’s could be either undescribed species, known species not present in databases or not identifiable at species level with rDNA markers. On the other hand, the NGS techniques are highly sensitive and return a large number of sequences that, if correctly trimmed and filtered, produce large amounts of information. Furthermore, the adoption of a phylogenetic approach, in addition to the sequences blast to database, helps in allocating the OTU in a tree to the most probable cluster (i.e. a fungal order, genus or even species). It is obvious that NGS data must be carefully analysed and critically evaluated in an ecological and epidemiological context. However, the adoption of a NGS strategy to sentinel nurseries can find most of the microbiota biodiversity associated with the samples, provide evidence of new species and reveal cryptic, potentially pathogenic taxa with endophytic behaviour that may be introduced unnoticed with native hosts.

From the results of NGS analyses, and considering the 18 OTU’s identified at species level, it becomes apparent how fungi, regardless their lifestyle, can potentially move unnoticed with plants not previously recorded as hosts.

Eight out of eighteen fungal species identified were not previously recorded in Europe (Table 1). Among these, five are described as plant pathogens, while *C*. *yunnanense* and the two *Paraconiothyrium* species are described as endophytes, although these two genera include several plant pathogenic species [37].

Four taxa, in addition to *P*. *foliicola*, deserve particular consideration for the risk they might pose to European woody species if introduced.

*Colletotrichum yunnanense* from *B*. *microphylla* is an Asian species recorded and described in China as an endophyte of an undetermined *Buxus* sp. [38]. The genus *Colletotrichum* (Sordariomycetes, Ascomycota) comprises ~600 species attacking over 3,200 species of monocot and dicot plants [23]. However, phytopathogenic species from this genus are capable of mutualistic or commensal lifestyles on plants other than those in which they cause disease [39]. Different *Colletotrichum* species have been found in endophytic fungal communities of a large variety of plants from different ecosystems, such as *Vigna unguiculata* (Fabaceae), *Taxus mairei* (Taxaceae), *Coffea arabica* (Rubiaceae), *Camptotheca acuminate* (Nyssaceae) and *Jatropha curcas* (Euphorbiaceae) [40]. A direct or indirect (through differential evolution and/or hybridization) host jump from native to exotic host could result in the shift from a commensal or low pathogenic behaviour, to a high pathogenic one. Because *Buxus microphylla* is the Asian *Buxus* species that is most often traded to Europe, the potential pathogenic behaviour of *C*. *yunnanense* when exposed to *B*. *sempervirens* should be assessed on a precautionary basis, PRA carried out and, if deemed necessary, measures to mitigate the risk of introduction developed and implemented.

*Phyllosticta citrichinaensis* is a leaf pathogen of *Citrus* spp. recorded only in China [41]. It induces small, grey-brown protuberant freckles on leaves of mandarin, red-brown protuberant freckles on leaves of orange, and small sunken, grey-brown spots with a dark brown margin and olive-green halos on leaves of pomeloes [41]. In this study, ITS sequences related to those of *P*. *citrichinaensis* (97% identity) have been recorded on *F*. *chinensis* and, to a lesser extent, *B*. *microphylla*, which clustered, distinctly from *P*. *citrichinaensis*, but within a well-supported unique clade. Whether they indicate variation within *P*. *citrichinaensis* or a distinct taxon is difficult to assess at this stage. In fact, NGS is a perfect tool for broad-spectrum screening of environmental samples but in order to identify samples at species level, it must be integrated with additional methods such as culturing or specific primers for PCR analysis.

*Phyllactinia populi* on *F*. *chinensis* deserves special consideration. This is a powdery mildew biotroph found on a large number of *Populus* species in Asia only, mostly in China [23]. Among the hosts there are also some European species such as *Populus nigra*. *Fraxinus chinensis* is apparently a non-host of *P*. *populi*, and the detection of the fungus as sequence could simply indicate external contamination. It cannot be excluded, however, that living inoculum could move with a non-host species through trading both as external contamination or as asymptomatic infection. For example, it has been suggested that the oak powdery mildew *Erysiphe quercicola* was introduced to Europe with two tropical hosts commonly traded to Europe as ornamentals, *Cinnamomum camphora* and *Murraya paniculata* [7]. Therefore, the risk of introduction of an alien pathogen as contaminant should not be neglected. Recognition of external contamination as a possible way for introduction must be taken in consideration in the detection protocols for ‘sentinel nursery’ plants, specifically in the recommendation for samples disinfection. The spray of plants with broad-spectrum contact fungicides, such as copper-based ones, should be recommended at time of delivery from exporting country and/or arrival at the importing country.

*Spencermartinsia viticola*, already recorded as a pathogen in different regions of the world, was detected asymptomatically on *I*. *cornuta*. The species belongs to the Botryosphaeriaceae complex that is responsible for trunk disease of grapevine worldwide [42]. *Spencermartinsia viticola* was first described in Spain on grapevine in 2005 [43] and has since been recorded in several countries around the world [44], which suggests rapid movement possibly through the trade of live plants. Furthermore, *S*. *viticola* is known as a pathogen of *Citrus* spp. (Rutaceae) in California [45], of *Eriobotrya japonica* (Rosaceae) in Spain [46] and *Prunus* spp. (Rosaceae) and other natural and domesticated woody species in South Africa [47] [48]. In China *S*. *viticola* was reported on *Populus cathayana* (Salicaceae) as first record for the country [49].

One-third of the total number of OTU’s (38) were found associated with *F*. *chinensis*. Twelve out of eighteen, and five out of six of the OTU’s identified at species level by NGS and culturing respectively, were also associated with *F*. *chinensis* suggesting this species is an important potential pathway of introduction of alien fungi to Europe. European ash species are actually suffering from severe dieback caused by the alien invasive *Hymenoscyphus fraxineus* (T. Kowalski) Baral, Queloz & Hosoya [4]. In Asia, *H*. *fraxineus* has been recorded on *F*. *chinensis* on which it is apparently hemi-biotrophic and does not cause disease [50] suggesting *F*. *chinensis* is one of the possible pathways for *H*. *fraxineus* movement through trade of living plants.

Results of this study also provide a more than speculative indication of how some disease agents can be introduced using pathways other than co-generic hosts. For example, import of *Citrus* and *Vitis* plants and propagation material is prohibited, or strictly regulated, in several countries around the world because of the risk of introduction of alien disease agents on such important crops. Movement of potential pathogenic species on hosts different from the known host highlights the need of research on alternative pathways of introduction of alien invasive plant pathogens of important crops, as suggested by Eschen et al. [51] for *Citrus*. While clearly a powerful tool, some constraints have to be solved to make a ‘sentinel nursery’ strategy applicable as a routine quarantine procedure. In order to avoid unintentional introductions, all the plant samples should be processed at the area of origin of the sentinel plants according to a common protocol that aims to identify species of most microorganisms detected. In this regard, the NGS protocols should be developed in order to guarantee a multiple marker analysis. In fact, the use of single markers (e.g. rDNA) cannot guarantee the identifications of OTUs at species level. The enrichment of the existing databases with fungal sequences, specifically of those loci still not well represented, is also necessary. Finally, a panel of plant species, native to the importing country, should be chosen and used for screening of susceptibility to the potential pathogenic species identified in the sentinel nurseries at the exporting countries.

## Conclusion

Sentinel nurseries represent a potential answer to address the lack of knowledge on: i) plant pests and pathogens in countries from where live plants are traded; ii) alternate pathways of introduction of pests and pathogens potentially harmful to forestry, landscape and crop trees in the importing country.

The sentinel nursery concept strictly applies to the evaluation of risk associated with pathways for pest and pathogens introduction and does not provide an indication of the potential for damage to plants in the imported country.

The implementation of sentinel nurseries combined with plantation of a selection of tree species, that are deemed important to the importing country (sentinel plantations) [9, 10] would enable the assessment of damage to protection goals for the importing country. This combination of sentinel nurseries and sentinel plantations might, if widely applied, contribute to a decrease in the risk of movement and introduction of IFP worldwide.

## Supporting information

S1 Table Fungal species used in phylogenetic analysis and accession number of ITS and GAPDH sequences. (DOCX)

S1 Fig Neighbour-joining trees based on ITS (A and C) and concatenated ITS and gdp (B) sequences of isolates of *Diaporthe liquidambaris*, *Diaporthe capsici*, *Nothophoma quercina*, *Botryosphaeria dothidea* and *Alternaria alternata*. Numbers above branches represent bootstrap support for the nodes and posterior probability based on Bayesian analysis of the dataset (in bold).
(TIF)

S2 Fig Neighbour-joining trees of the 17 OTUs resolved at species level (known or putative new species), with the exception of the OTU82 (Fig 3). Each tree includes the OTU sequence and the ITS sequence of the top BLAST hits species (with GenBank accession numbers). Numbers above branches represent bootstrap support for the nodes and posterior probability based on Bayesian analysis of the dataset (in bold).
(PPT)
