# Supplementary material for: The sentinel tree nursery as an early warning system for pathway risk assessment: Fungal pathogens associated with Chinese woody plants commonly shipped to Europe
Source: PLoS One. 2017 Nov 29;12(11):e0188800. doi: 10.1371/journal.pone.0188800 (PMC5706704; doi:10.1371/journal.pone.0188800)
Supplement: S1 Table — (DOCX) [file pone.0188800.s001.docx]

**S1 Table. Fungal species used in phylogenetic analysis and accession number of ITS and GAPDH sequences.**

| Species | accession number | |
| --- | --- | --- |
|  | ITS | GAPDH |
| *Alternaria alternata* (this study) | KY865309 | KY865308 |
| *Alternaria alternata strain CBS 102599* | KP124185 | KP124330 |
| *Alternaria alternata strain CBS 107.53* | KP124162 | KP124305 |
| *Alternaria alternata strain CBS 620.83* | KP124171 | KP124315 |
| *Alternaria alternata strain CBS 795.72* | KP124166 | KP124309 |
| *Alternaria burnsii strain CBS 118816* | KP124273 | KP124423 |
| *Alternaria jacinthicola strain CBS 878.95* | KP124286 | KP124437 |
| *Botryosphaeria dothidea* | AY236947 |  |
| *Blumeria graminis* f. sp. *poe* | AJ313145 |  |
| *Blumeria graminis* | AB273565 |  |
| *Blumeria graminis* | KF545644 |  |
| *Blumeria graminis* f. sp. *avenae* | AF011284 |  |
| *Blumeria graminis* f. sp. *hordei* | HM484333 |  |
| *Blumeria graminis* f. sp. *secalis* | HM484331 |  |
| *Blumeria graminis* f. sp. *tritici* | HM484334 |  |
| *Botryosphaeria dothidea* | AY236947 |  |
| *Botryosphaeria dothidea* | AF241175 |  |
| *Botryosphaeria dothidea* | AY236950 |  |
| *Botryosphaeria dothidea* (this study) | KY865310 |  |
| *Botryosphaeria dothidea* | AY236948 |  |
| *Botryosphaeria dothidea* | AF027751 |  |
| *Chaetopyrena penicillata* | KY681683 |  |
| *Chaetopyrena penicillata* | KC492443 |  |
| *Chaetopyrena penicillata* | JQ663990 |  |
| *Cistella acuum* | GU727552 |  |
| *Colletotrichum dracaenophilum* | KJ186110 |  |
| *Colletotrichum dracaenophilum* | KU612894 |  |
| *Colletotrichum truncatum* | KU985044 |  |
| *Colletotrichum truncatum* | KU985045 |  |
| *Colletotrichum yunnanense* | NR137098 |  |
| *Colletotrichum yunnanense* | JX546804 |  |
| *Cryptococcus carnescens* | EU149785 |  |
| *Cryptococcus carnescens* | EU149785 |  |
| *Cryptococcus tephrensis* | JX188130 |  |
| *Cryptococcus tephrensis* | JX188132 |  |
| *Cryptococcus tephrensis* | JX188134 |  |
| *Diaporthe helianthi* | KM979994 |  |
| *Diaporthe helianthi* | KM979782 |  |
| *Diaporthe capsici* | KR870865 |  |
| *Diaporthe capsici* | KR870864 |  |
| *Diaporthe capsici* | KR870863 |  |
| *Diaporthe capsici* (this study) | KY947551 |  |
| *Diaporthe liquidambaris* | KU751869 |  |
| *Diaporthe liquidambaris* | FJ478124 |  |
| *Diaporthe liquidambaris* | HQ328002 |  |
| *Diaporthe liquidambaris* (this study) | KY865313 |  |
| *Diaporthe liquidambaris* | KX866890 |  |
| *Diaporthe phyllanthicola* | KF498871 |  |
| *Dioszegia zsoltii var. yunnanensis* | AF385449 |  |
| *Dioszegia zsoltii* | EU070921 |  |
| *Dioszegia catarinonii* | AY562154 |  |
| *Dioszegia catarinonii* | KY103349 |  |
| *Dioszegia takashimae* | NR136971 |  |
| *Dioszegia takashimae* | DQ003332 |  |
| *Dioszegia athyri* | EU070926 |  |
| *Dioszegia antarctica* | KY103342 |  |
| *Dioszegia crocea* | AJ581075 |  |
| *Dioszegia aurantiaca* | EU266497 |  |
| *Dothiorella acacicola* | JX014406 |  |
| *Dothiorella capri-amissi* | NR136996 |  |
| *Dothiorella longicollis* | NR136999 |  |
| *Dothiorella longicollis* | KF766162 |  |
| *Epicoccum nigrum* | MF034106 |  |
| *Epicoccum nigrum* | KX099630 |  |
| *Epicoccum sorghinum* | KT310094 |  |
| *Epicoccum sorghinum* | KT310095 |  |
| *Erysiphe magnoliicola* | KJ567073 |  |
| *Erythrobasidium hasegawianum* | NR111008 |  |
| *Erythrobasidium hasegawianum* | AF444522 |  |
| *Erythrobasidium hasegawianum* | KT809055 |  |
| *Erythrobasidium yunnanense* | KY103394 |  |
| *Erythrobasidium yunnanense* | FJ515217 |  |
| *Erythrobasidium elongatum* | KY103392 |  |
| *Escovopsis weberi* | KX963338 |  |
| *Infundichalara microchona* | KF156300 |  |
| *Infundichalara microchona* | HM036588 |  |
| *Infundichalara microchona* | KF359590 |  |
| *Leptosphaerulina chartarum* | GU073119 |  |
| *Leptosphaerulina chartarum* | KP132534 |  |
| *Leptosphaerulina chartarum* | KP132534 |  |
| *Leptosphaerulina chartarum* | KP132535 |  |
| *Leptosphaerulina americana* | AY278318 |  |
| *Leptosphaerulina arachidicola* | KJ612070 |  |
| *Leptosphaerulina trifolii* | AY131203 |  |
| *Leucosporidium creatinivorum* | KJ708418 |  |
| *Leucosporidium scottii* | KY104032 |  |
| *Leucosporidium scottii* | KY104033 |  |
| *Leucosporidium drummii* | FN908919 |  |
| *Leucosporidium escuderoi* | JN197600 |  |
| *Myrothecium verrucaria* | EF211127 |  |
| *Myrothecium macrosporum* | KP744448 |  |
| *Neofusicoccum ribis* | AF241177 |  |
| *Neofusicoccum ribis* | AF027743 |  |
| *Nothophoma quercina* | KU973714 |  |
| *Nothophoma quercina* | KX645664 |  |
| *Nothophoma quercina (this study)* | KY865312 |  |
| *Nothophoma quercina* | KT037421 |  |
| *Paraconiothyrium brasiliense* | KF574893 |  |
| *Paraconiothyrium brasiliense* | JX496044 |  |
| *Paraconiothyrium brasiliense* | KM520128 |  |
| *Paraconiothyrium hawaiiense* | EU715661 |  |
| *Paraconiothyrium hawaiiense* | HM751092 |  |
| *Paraconiothyrium archidendri* | KY327411 |  |
| *Paraconiothyrium archidendri* | KY327412 |  |
| *Paraconiothyrium fuckelii* | KJ939278 |  |
| *Paraconiothyrium fuckelii* | JX496096 |  |
| *Paraconiothyrium fuckelii* | JX496112 |  |
| *Paraphoma radicina* | KX784240 |  |
| *Paraphoma radicina* | KX784241 |  |
| *Paraphoma radicina* | KX784243 |  |
| *Paraphoma vinacea* | KU176886 |  |
| *Paraphoma vinacea* | KU176887 |  |
| *Paraphoma chrysanthemicola* | GU395507 |  |
| *Phoma* sp. | KT989560 |  |
| *Phoma herbarum* | JX421724 |  |
| *Phoma herbarum* | JX421725 |  |
| *Phoma herbarum* | KT004577 |  |
| *Phyllactinia antarctica* | AB080535 |  |
| *Phyllactinia fraxini* | AB080552 |  |
| *Phyllactinia populi* | KC357772 |  |
| *Phyllactinia populi* | JQ250805 |  |
| *Phyllactinia populi* | JF795489 |  |
| *Phyllosticta* sp. 39-4-1 | JX014406 |  |
| *Phyllosticta aloeicola* | KR183768 |  |
| *Phyllosticta citrichinaensis* | JN791598 |  |
| *Phyllosticta citrichinaensis* | JN791611 |  |
| *Pseudocosmospora vilior* | GU726755 |  |
| *Pseudonectria buxi* | KM231778 |  |
| *Pseudonectria buxi* | KM231779 |  |
| *Pseudonectria buxi* | HQ897800 |  |
| *Pseudonectria foliicola* | KM231776 |  |
| *Pseudonectria foliicola* (this study) | KY865314 |  |
| *Pseudonectria buxi* | JF937563 |  |
| *Pseudonectria rousseliana* | JF937564 |  |
| *Spencermartinsia viticola* | KM103229 |  |
| *Volutella buxi* | JX535311 |  |
| *Xenochalara juniperi* | DQ093775 |  |
| *Xenopolyscytalum pinea* | HQ599580 |  |
|  |  |  |
