# Supplementary figures and images for: The sentinel tree nursery as an early warning system for pathway risk assessment: Fungal pathogens associated with Chinese woody plants commonly shipped to Europe
Source: PLoS One. 2017 Nov 29;12(11):e0188800. doi: 10.1371/journal.pone.0188800 (PMC5706704; doi:10.1371/journal.pone.0188800)

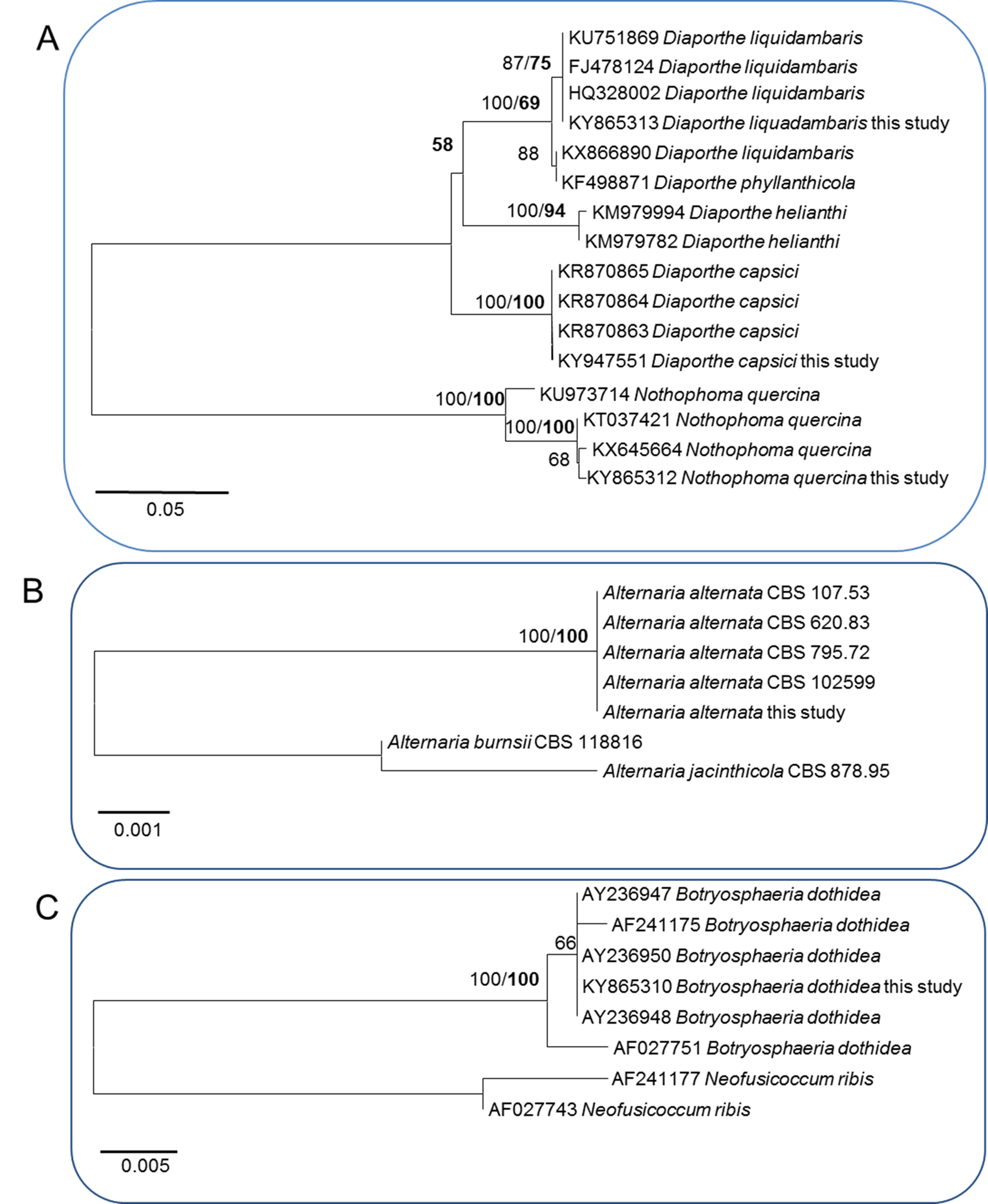

Supplement: S1 Fig — Numbers above branches represent bootstrap support for the nodes and posterior probability based on Bayesian analysis of the dataset (in bold). (TIF) [file pone.0188800.s002.tif]
