# Supplementary material for: The sentinel tree nursery as an early warning system for pathway risk assessment: Fungal pathogens associated with Chinese woody plants commonly shipped to Europe
Source: PLoS One. 2017 Nov 29;12(11):e0188800. doi: 10.1371/journal.pone.0188800 (PMC5706704; doi:10.1371/journal.pone.0188800)
Supplement: S2 Fig — Each tree includes the OTU sequence and the ITS sequence of the top BLAST hits species (with GenBank accession numbers). Numbers above branches represent bootstrap support for the nodes and posterior probability based on Bayesian analysis of the dataset (in bold). (PPT) [file pone.0188800.s003.ppt]

## Slide 1
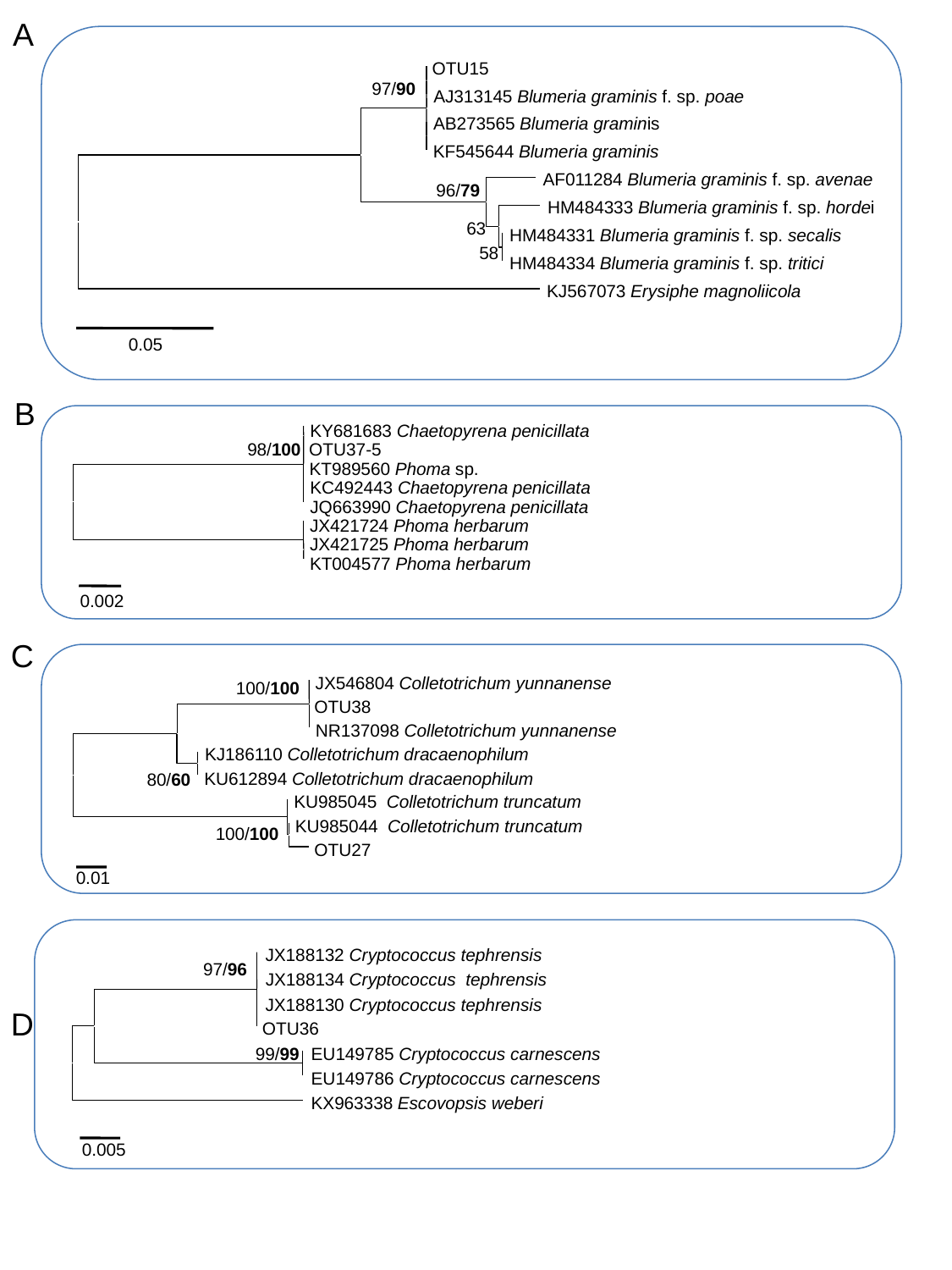

A
 OTU15
97/90
 AJ313145 Blumeria graminis f. sp. poae
 AB273565 Blumeria graminis
 KF545644 Blumeria graminis
 AF011284 Blumeria graminis f. sp. avenae
96/79
 HM484333 Blumeria graminis f. sp. hordei
63
 HM484331 Blumeria graminis f. sp. secalis
58
 HM484334 Blumeria graminis f. sp. tritici
 KJ567073 Erysiphe magnoliicola
0.05
B
 KY681683 Chaetopyrena penicillata
98/100
 OTU37-5
 KT989560 Phoma sp.
 KC492443 Chaetopyrena penicillata
 JQ663990 Chaetopyrena penicillata
 JX421724 Phoma herbarum
 JX421725 Phoma herbarum
 KT004577 Phoma herbarum
0.002
C
 JX546804 Colletotrichum yunnanense
100/100
 OTU38
 NR137098 Colletotrichum yunnanense
 KJ186110 Colletotrichum dracaenophilum
 KU612894 Colletotrichum dracaenophilum
80/60
 KU985045 Colletotrichum truncatum
 KU985044 Colletotrichum truncatum
 OTU27
0.01
100/100
 JX188132 Cryptococcus tephrensis
97/96
 JX188134 Cryptococcus tephrensis
 JX188130 Cryptococcus tephrensis
 OTU36
99/99
 EU149785 Cryptococcus carnescens
 EU149786 Cryptococcus carnescens
 KX963338 Escovopsis weberi
0.005
D

## Slide 2
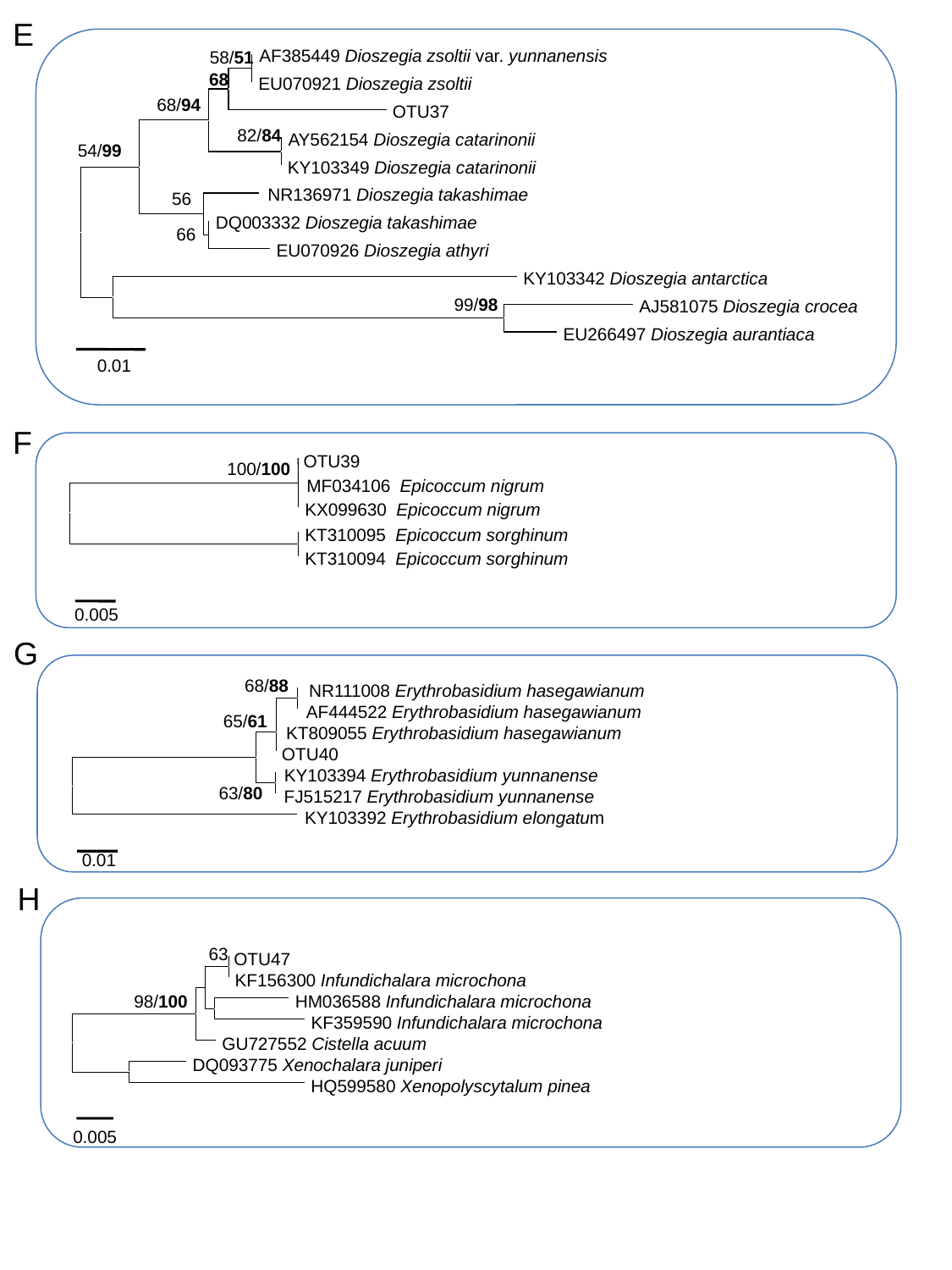

E
 AF385449 Dioszegia zsoltii var. yunnanensis
58/51
 EU070921 Dioszegia zsoltii
68/94
 OTU37
82/84
 AY562154 Dioszegia catarinonii
54/99
 KY103349 Dioszegia catarinonii
 NR136971 Dioszegia takashimae
56
 DQ003332 Dioszegia takashimae
66
 EU070926 Dioszegia athyri
 KY103342 Dioszegia antarctica
99/98
 AJ581075 Dioszegia crocea
 EU266497 Dioszegia aurantiaca
0.01
68
F
 OTU39
100/100
 MF034106 Epicoccum nigrum
 KX099630 Epicoccum nigrum
 KT310095 Epicoccum sorghinum
 KT310094 Epicoccum sorghinum
0.005
G
68/88
 NR111008 Erythrobasidium hasegawianum
 AF444522 Erythrobasidium hasegawianum
65/61
 KT809055 Erythrobasidium hasegawianum
 OTU40
 KY103394 Erythrobasidium yunnanense
63/80
 FJ515217 Erythrobasidium yunnanense
 KY103392 Erythrobasidium elongatum
0.01
H
63
 OTU47
 KF156300 Infundichalara microchona
98/100
 HM036588 Infundichalara microchona
 KF359590 Infundichalara microchona
 GU727552 Cistella acuum
 DQ093775 Xenochalara juniperi
 HQ599580 Xenopolyscytalum pinea
0.005

## Slide 3
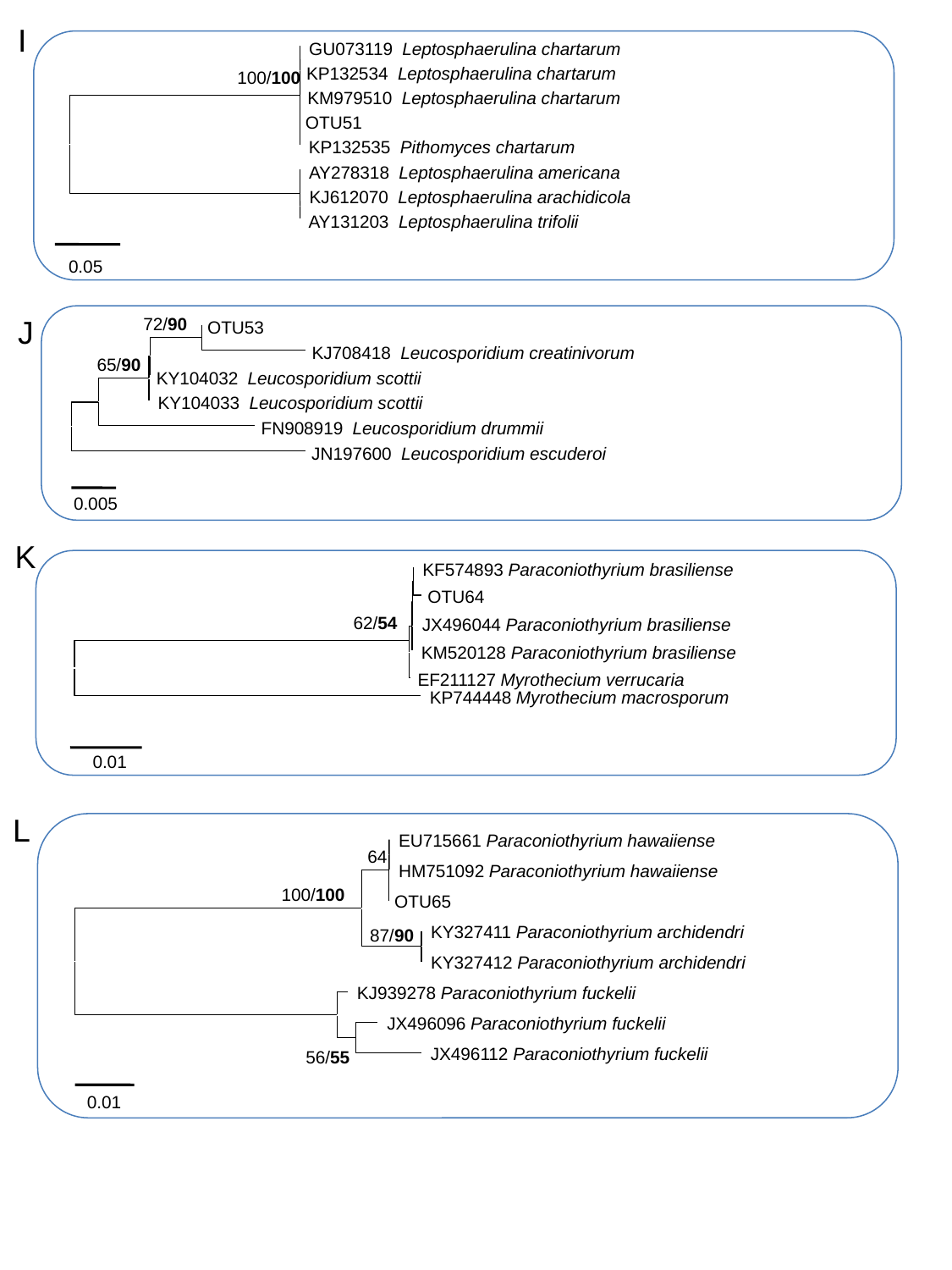

I
 GU073119 Leptosphaerulina chartarum
 KP132534 Leptosphaerulina chartarum
100/100
 KM979510 Leptosphaerulina chartarum
 OTU51
 KP132535 Pithomyces chartarum
 AY278318 Leptosphaerulina americana
 KJ612070 Leptosphaerulina arachidicola
 AY131203 Leptosphaerulina trifolii
0.05
72/90
 OTU53
 KJ708418 Leucosporidium creatinivorum
65/90
 KY104032 Leucosporidium scottii
 KY104033 Leucosporidium scottii
 FN908919 Leucosporidium drummii
 JN197600 Leucosporidium escuderoi
0.005
J
K
 KF574893 Paraconiothyrium brasiliense
 OTU64
62/54
 JX496044 Paraconiothyrium brasiliense
 KM520128 Paraconiothyrium brasiliense
 EF211127 Myrothecium verrucaria
 KP744448 Myrothecium macrosporum
0.01
L
 EU715661 Paraconiothyrium hawaiiense
64
 HM751092 Paraconiothyrium hawaiiense
100/100
 OTU65
 KY327411 Paraconiothyrium archidendri
87/90
 KY327412 Paraconiothyrium archidendri
 KJ939278 Paraconiothyrium fuckelii
 JX496096 Paraconiothyrium fuckelii
 JX496112 Paraconiothyrium fuckelii
56/55
0.01

## Slide 4
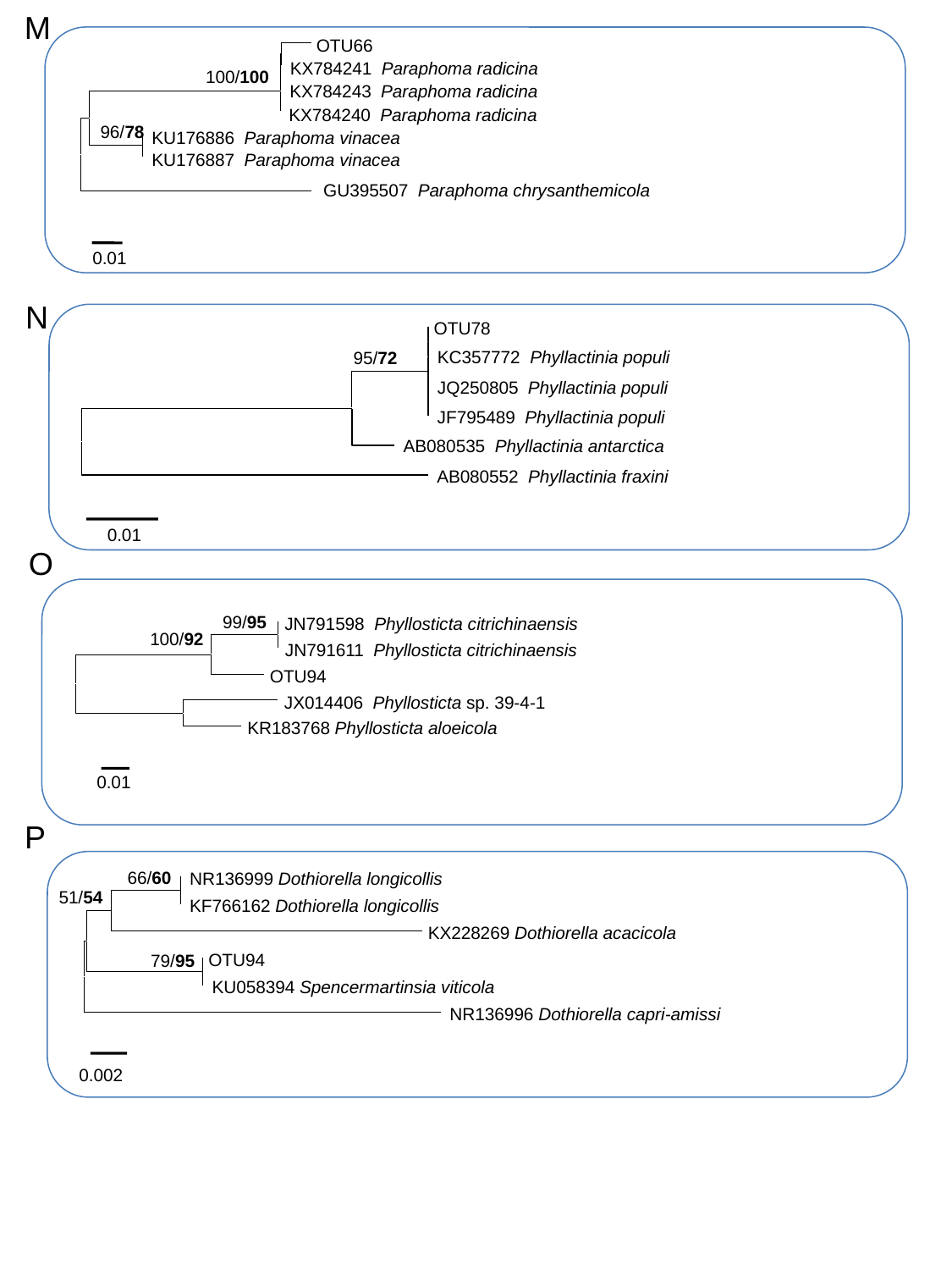

M
 OTU66
 KX784241 Paraphoma radicina
100/100
 KX784243 Paraphoma radicina
 KX784240 Paraphoma radicina
96/78
 KU176886 Paraphoma vinacea
 KU176887 Paraphoma vinacea
 GU395507 Paraphoma chrysanthemicola
0.01
N
 OTU78
 KC357772 Phyllactinia populi
95/72
 JQ250805 Phyllactinia populi
 JF795489 Phyllactinia populi
 AB080535 Phyllactinia antarctica
 AB080552 Phyllactinia fraxini
0.01
O
99/95
 JN791598 Phyllosticta citrichinaensis
100/92
 JN791611 Phyllosticta citrichinaensis
 OTU94
 JX014406 Phyllosticta sp. 39-4-1
 KR183768 Phyllosticta aloeicola
0.01
P
66/60
 NR136999 Dothiorella longicollis
51/54
 KF766162 Dothiorella longicollis
 KX228269 Dothiorella acacicola
 OTU94
79/95
 KU058394 Spencermartinsia viticola
 NR136996 Dothiorella capri-amissi
0.002
